# Tuberculosis-Induced Immune-Mediated Necrotizing Myopathy: A Challenging Case Scenario in a Non-Endemic Country

**DOI:** 10.3390/reports7040082

**Published:** 2024-09-24

**Authors:** Agnese Colpani, Davide Astorri, Andrea De Vito, Giordano Madeddu, Sandro Panese, Nicholas Geremia

**Affiliations:** 1 Unit of Infectious Diseases, Department of Medicine, Surgery and Pharmacy, University of Sassari, 07100 Sassari, Italy; giordano@uniss.it; 2 Rheumatology Unit, Department of Medicine, Ospedale Civile “S.S. Giovanni e Paolo”, 30122 Venice, Italy; davide.astorri@aulss3.veneto.it; 3 School in Biomedical Science, Biomedical Science Department, University of Sassari, 07100 Sassari, Italy; 4 Unit of Infectious Diseases, Department of Clinical Medicine, Ospedale “dell’Angelo”, 30174 Venice, Italy; sandro.panese@aulss3.veneto.it (S.P.); nicholas.geremia@aulss3.veneto.it (N.G.); 5 Unit of Infectious Diseases, Department of Clinical Medicine, Ospedale Civile “S.S. Giovanni e Paolo”, 30122 Venice, Italy

**Keywords:** tuberculosis, immune-mediated necrotizing myopathy, anti-HMGCR myopathy

## Abstract

**Background and Clinical Significance**: Tuberculosis (TB) poses a significant global health challenge; although low–middle income countries carry the heaviest burden, its diagnosis and treatment can be challenging in any country. The clinical picture can be complex and vary from person to person, with autoimmune complications that can hinder TB diagnosis and treatment. **Case Presentation**: We report the case of a 38-year-old man from Bangladesh who had recently arrived in Italy through the Balkan route. He presented with TB in the cervical lymph nodes and long-standing chronic myalgias. While a wide range of TB-triggered autoimmune entities can be found in the literature, this case is the first to describe immune-mediated necrotizing myopathy (IMNM) triggered by active TB. **Conclusions**: IMNM has been previously associated only with other infections like SARS-CoV-2 and Dengue. The successful diagnosis and management of TB-induced IMNM was achieved through a collaborative, multidisciplinary approach involving rheumatologists, immunologists, and infectious diseases specialists, showcasing an innovative treatment strategy and adding new insights into the complexities of TB and IMNM.

## 1. Introduction and Clinical Significance

Tuberculosis (TB), caused by the bacterium *Mycobacterium tuberculosis*, remains one of the most significant infectious diseases globally, infecting approximately 10 million people each year [1]. Despite extensive public health efforts, TB continues to pose a severe threat, particularly in low- and middle-income countries where healthcare resources are often limited [2,3]. TB is not only a disease of the lungs but can affect any organ system, leading to a wide range of clinical manifestations that complicate its diagnosis and treatment [4,5].

While TB is primarily known for its pulmonary implications, its potential to trigger or exacerbate a variety of immune-mediated diseases is increasingly recognized. The intricate interplay between the mycobacterial antigens and the host’s immune system can lead to autoimmune responses [1]. These responses, while aimed at controlling the infection, can inadvertently harm the host [6]. Such autoimmune phenomena can exacerbate the disease course, leading to challenging complications in clinical settings.

Immune-mediated necrotizing myopathy (IMNM) is characterized by acute or subacute muscle weakness, severe pain, and elevated creatine kinase (CPK) [7,8]. At the biopsy, it is associated with myofiber necrosis with minimal inflammatory cell infiltration on muscle; extra-muscular involvement is uncommon [8]. While commonly associated with the use of statins, IMNM can also follow infections and vaccinations, highlighting the body’s complex immune response to different triggers [9,10,11,12]. However, the association of IMNM with TB is rare and poorly documented, making cases of TB-induced IMNM particularly noteworthy.

This paper presents a unique case of IMNM following an active tuberculosis infection in a 38-year-old man, which, to our knowledge, is the first of its kind. This case underscores the complex interactions between infectious diseases and immune-mediated conditions and highlights the need for heightened awareness and a multidisciplinary approach in similar clinical scenarios. By exploring this case, we aim to add valuable insights into the lesser-known implications of TB and expand the understanding of its potential to induce or trigger immune-mediated diseases, contributing to the broader field of infectious diseases and immunology.

## 2. Case Presentation

At the end of January 2024, a 38-year-old male from Bangladesh presented to the emergency room complaining of pharyngodynia and worsening neck swelling. He had arrived in Italy six months before, having travelled via the Balkan route; this path is frequently used by migrants from Asia to reach Western Europe. This route is characterized by prolonged journeys under harsh conditions, overcrowded living spaces, inadequate sanitation, and limited access to healthcare services. These factors significantly increase exposure to infectious diseases, including TB, and the risk of reactivating latent infections due to stress, malnutrition, and compromised immunity [13,14,15].

He denied any previous medical history.

**Figure 1:**
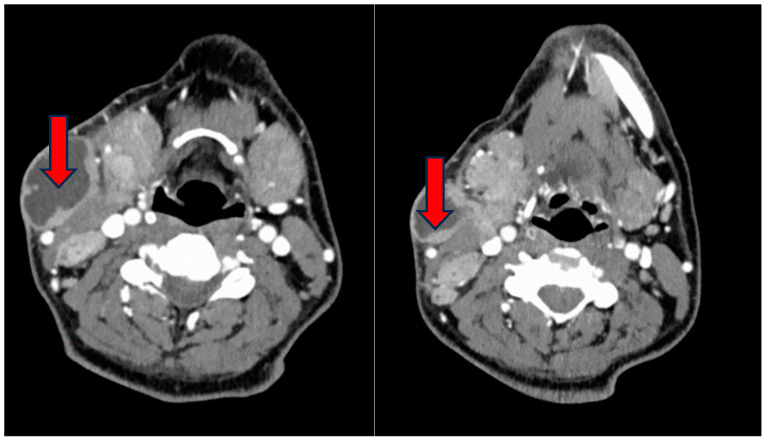
Neck CT scan confirming a conglomerated centrally colliquated mass (red arrows), extending anteriorly and posteriorly to the sternocleidomastoid muscle of 8 × 4.4 × 10 cm.

During the physical examination, a notable swelling was observed on the left side of the neck, exhibiting characteristics of softness and tenderness upon palpation, with a diameter of at least 10 cm. Furthermore, the examination revealed hypertrophy and erythema of the right tonsil, notable for the absence of exudate. A neck ultrasound was performed, revealing the presence of two colliquated lymph nodes in the right lateral-cervical region. Subsequent CT imaging confirmed the existence of a conglomerated centrally colliquated mass, extending anteriorly and posteriorly to the sternocleidomastoid muscle, measuring 8 × 4.4 × 10 cm (Figure 1). Additionally, a second lesion was identified in the posterior lateral-cervical homolateral region.

A chest X-ray was conducted, indicating no abnormalities. However, the QuantiFERON test returned positive. Thus, he was admitted to the Infectious Diseases ward for further investigations.

**Figure 2:**
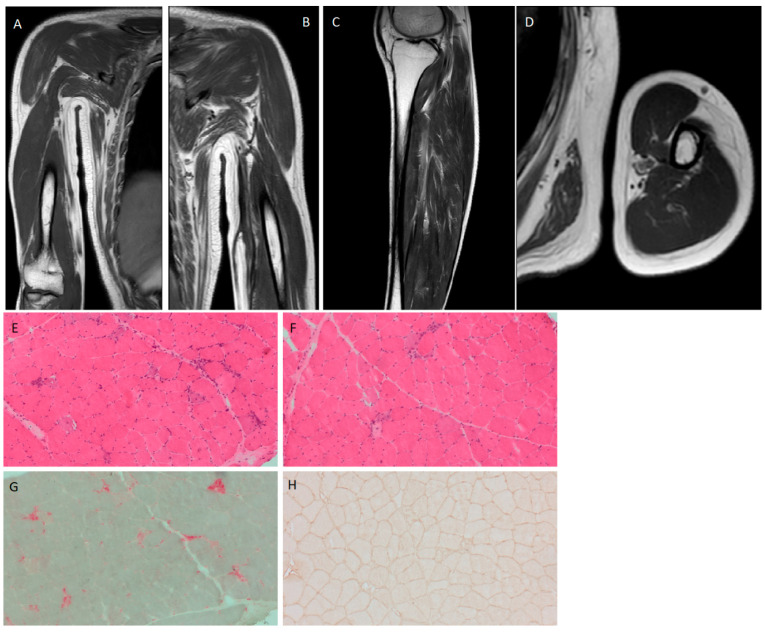
MRI showing diffuse edema of the arms bilaterally (**A**,**B**), thighs (**C**), and gluteus (**D**), with initial atrophy; muscle biopsy highlighting moderate necrotic myositis; (**E**,**F**): haematoxylin-eosin, (**G**): acid phosphatase, (**H**): Major Histocompatibility Complex-I.

During the hospitalization, he was evaluated by an otolaryngologist who performed a fine needle aspiration of the enlarged lymph nodes and a biopsy of the right tonsil. Direct microscopic examination with Ziehl–Neelsen staining revealed the presence of the acid-fast bacilli; the presence of *M. tuberculosis* complex was later confirmed by Xpert^®^ MTB/RIF and liquid culture (BACTEC MGIT 960 system). Notably, Ziehl–Neelsen staining of the tonsil biopsy sample returned negative. Direct examination and culture of sputum, urine, and feces were negative for TB; HIV screening was also negative. Consequently, on the 1st of February, the patient was initiated on a four-drug antituberculosis regimen with isoniazid 300 mg/daily plus rifampicin 600 mg/daily plus pyrazinamide 1000 mg/daily plus ethambutol 1200 mg/daily. After a few days of admission, the patient reported long-standing myalgias and weakness in both the inferior and superior extremities. Laboratory tests revealed significant levels of creatine phosphokinase (CPK) at 12,354 U/L (normal range: 30–240 U/L), along with elevated levels of myoglobin and aldolase. Further findings are reported in Table 1. After a collegial discussion with rheumatologists, he underwent magnetic resonance imaging (MRI) scans of the legs and arms. This showed diffuse edema affecting the gluteus, thighs, and all the arm muscles bilaterally, accompanied by initial atrophy (Figure 2).

Subsequent electromyography evidenced signs of discrete primary muscular suffering with abundant voluntary activity in all areas examined.

A muscle biopsy was performed and showed variability in the diameter of muscle fibers due to isolated atrophic fibers with Gomori’s trichromic and hematoxylin-eosin staining. In addition, cell necrosis, some phagocytosis, and many degenerated fibers were observed, as well as aspects of vacuolization. There were no inflammatory infiltrates, but connective tissue around rare fibers was found. Moreover, normal differentiation and distribution of fiber types, with atrophic fibers predominantly of type 1 and 2B and the presence of type 2C fibers, were observed. Alkaline phosphatase positivity in necrosis fibers and at vacuoles was also found. On immunohistochemistry, the membranes of most muscle fibers expressed the Major Histocompatibility Complex-I (MHC-I). In addition, the deposit of the Complement Terminal Complex was present on the membrane of some fibers. Most of the cytoplasm of muscle fibers tested positive for P62. The histological exams confirmed the presence of moderate immunomediated necrotic myositis (Figure 2).

Meanwhile, the autoimmunity panel resulted in positive tests for antibodies against 3-hydroxy-3-methylglutaryl-coenzyme A reductase (anti-HMGCR), with a value of 227.9 CU by Chemiluminescence Immunoassay (CLIA); the autoimmunity panel is reported in Table 2. Serology for trichinellosis was also negative. A rheumatological work-up led to a diagnosis of IMNM.

**Figure 3:**
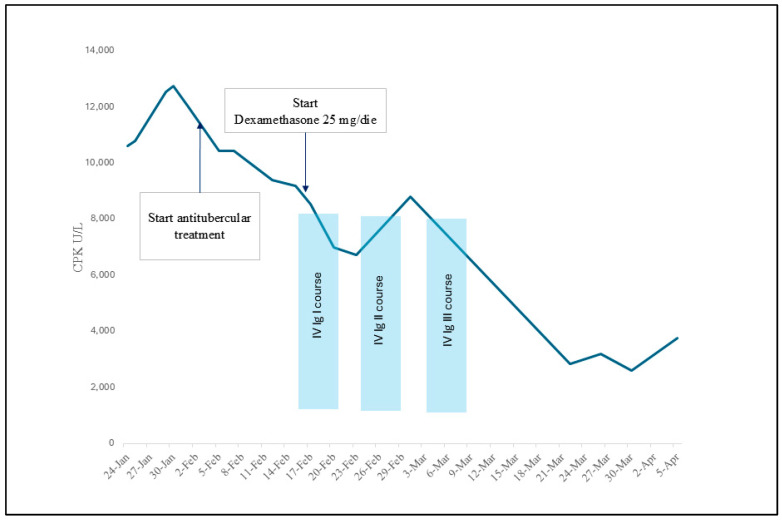
The creatine phosphokinase (CPK) trend in a patient with TB-induced immune-mediated necrotizing myopathy treated with dexamethasone 25 mg/die and intravenous immunoglobulins.

Due to active tuberculosis, aggressive immunosuppressive therapy was delayed, and on 16 February, the patient was started on dexamethasone 25 mg daily; also, he was started on a five-day course of intravenous immunoglobulins at the dosage of 0.4 g/Kg/daily, which was then repeated for a total of fifteen days. The follow-up showed a constant decrease in CPK with an improvement in myalgias (Figure 3); no further MRI images were collected. The susceptibility testing (BD Phoenix™, TBeXiST software) later revealed a resistance to pyrazinamide, which was then discontinued; the other first-line drugs were reported as sensitive. The test was conducted on the same sample collected from the lymph node lesion. The patient completed the antibiotic course as prescribed, with a four-drug regimen for the first two months, followed by four months of isoniazid and rifampicin, with the complete resolution of the neck lesion and a subjective improvement of the general condition.

## 3. Discussion

We presented the case of a 38-year-old man from Bangladesh admitted with a notable swelling of the neck and complaining of long standing myalgias, with no prior medical history. *M. tuberculosis* was identified on the sample collected from the colliquated nodes of the neck; a multidisciplinary approach led to the diagnosis of IMNM.

While definitive temporal causation cannot be established in a single case, the evidence suggests a plausible link between active, albeit initially undiagnosed, TB and the onset of IMNM in this patient. Also, the patient had no prior medical history and throughout investigations did not lead to any other possible trigger for INMN.

Immune-mediated necrotizing myopathy is a recently defined entity; anti-HMGCR myopathy was first described as linked to the use of statins. It usually debuts with progressive proximal muscular weakness, with highly elevated CPK levels [16]. A T1 hyperintensity, especially in the posterior thigh, is a common finding. The biopsy will show necrotizing myopathy with MHC-I or MAC staining. The electromyography will be consistent with a myopathic pattern, with spontaneous activity in the form of fibrillations and sharp waves. Anti-HMGCRs are highly specific and do not cross-react with other autoimmune conditions [16,17].

Despite most cases having been linked to statin exposure, several environmental factors have been hypothesized as possible causative triggers, including food, infections, and cancer. Patients’ genetic factors may also play a role [18,19,20,21,22].

Controlling the trigger is often not enough. Treatment is mainly based on corticosteroids with or without another immunosuppressive agent, and intravenous immunoglobulins. While treatment initiation is warranted as soon as possible, the best timing for tapering and subsequent withdrawal is less precise [23].

Muscle involvement during active TB may be due to different mechanisms. Muscular localization of *M. tuberculosis*, antituberculosis drug toxicity, and tuberculosis-induced autoimmune manifestations have been described so far.

Direct muscle involvement is rare, often due to contiguous spreading from affected bones or other sites. Isolated muscle TB is anecdotal; thus, it is usually burdened with a delayed diagnosis [24].

A second mechanism underlying muscle damage during TB is drug toxicity; data are scarce and mainly based on case reports. Of interest, rifampicin has been reported as a possible agent for drug-induced systemic lupus erythematous, which could lead to muscle involvement [25]. As for isoniazid, its role is certain, although with low risk [26]. While drug-induced lupus is often reversible after antibiotic withdrawal, a short course of corticosteroids may be required for complete resolution [27]. No other antituberculosis drug has been linked to muscle toxicity.

The third possible explanation for TB-induced muscle damage is autoimmunity. The complex interplay between infections and autoimmunity has been extensively narrated in the literature. Regarding TB, several mechanisms have been hypothesized for triggering autoimmunity processes; these include chronic infection, vitamin D deficiency, extensive cell death followed by inadequate clearance, enhanced activation of macrophages and dendritic cells, and genetic polymorphisms (of both host and bacteria) [1].

A wide array of autoantibodies has been associated with active TB [1]; nevertheless, the actual impact of the TB-induced autoimmunity process on patients’ health is yet to be clarified. Reports of autoimmunity-related events include erythema nodosum, erythema induratum, reactive arthritis (for example, Poncet’s disease), amyloidosis, fibromyalgia, scleritis, uveitis, tenosynovitis, and dactylitis [28,29,30].

Despite the absence of reported cases of TB-induced IMNM, we found reports of infection-induced IMNM following Dengue or SARS-CoV-2 [11,19]. Also, in our case, no other possible triggers were identified other than ongoing active TB. Thus, we considered the infection the most reasonable cause of the muscle injury. This diagnosis posed a significant treatment challenge. Aggressive and combined immunosuppressive therapy is warranted to treat IMNM. However, active TB forced us to redefine the treatment, tailoring it to the patient’s needs. He tolerated prednisone 25 mg and intravenous immunoglobulins, which were then repeated three times.

## 4. Conclusions

This case adds an exciting piece of information to the multifaceted clinical presentation of TB; in particular, we would like to highlight how challenging the treatment was of invalidating autoimmune-triggered comorbidities while treating active tuberculosis, posing the need for an individualized multidisciplinary approach involving rheumatologists, immunologists, and infectious disease specialists.

## Figures and Tables

**Table 1:** Laboratory findings at admission.

Indicator	Patient’s Value	Normal Value
WBC	11.5 × 10^3^/mmc	4.5–11 × 10^3^/mmc
RBC	4.49 × 10^6^/mmc	4.5–6.10 × 10^6^/mmc
HGB	12.6 g/dL	14–18 g/dL
PLT	330 × 10^3^/mmc	130–400 × 10^3^/mmc
Creatinine	0.54 mg/dL	0.72–1.18 mg/dL
ALT	278 U/L	1–45 U/L
LDH	932 U/L	25–248 U/L
CRP	28.6 mg/L	<5.0 mg/L
PCT	0.06 mcg/L	<0.5 mcg/L
CPK	12,354 U/L	10–171 U/L
Aldolase	83.4 U/L	0.0–7.6 U/L
Myoglobin	2329 ng/mL	17–106 ng/mL

WBC: white blood cells; RBC: red blood cells; HGB: hemoglobin; PLT: platelets; ALT: alanine aminotransferase; LDH: lactic dehydrogenases; CRP: C-reactive protein; PCT: procalcitonin; CPK: creatine phosphokinase.

**Table 2:** Autoimmunity panel.

Autoantibodies	Result
Anti-HMGCR	227.9 CU (n.v. < 20)
ANA on Hep-2	1:80, finely grained
ENA	NEGATIVE
AMA	NEGATIVE
ALKM	NEGATIVE
ASMA	NEGATIVE
Anti-dsDNA	0.60 UI/mL (n.v. < 10)
RF	<5.0 UI/mL (n.v. < 14)
C3	150 mg/dL (n.v. 90–180)
C4	47 mg/dL (n.v. 10–40)

ANA: anti-nuclear antibodies; ENA: extractable nuclear antigen; AMA: anti-mitochondrial antibodies; ALKM: anti-liver–kidney microsomal antibodies; ASMA: anti-smooth muscle antibodies; dsDNA: double stranded DNA; n.v.: normal value.

## Data Availability

The original data presented in the study are included in the article, further inquiries can be directed to the corresponding authors.
